# Optimization of lipid nanoparticles loaded with ribonucleoprotein-oligonucleotide complexes for *in vivo* delivery of a CRISPR/Cas9 system targeting hepatitis B virus

**DOI:** 10.1016/j.virusres.2025.199682

**Published:** 2025-12-24

**Authors:** Rupaly Akhter, Bouchra Kitab, Mohammad Enamul Hoque Kayesh, Rina Shimizu, Haruno Onuma, Naoki Yamamoto, Shintaro Ogawa, Masaya Sugiyama, Yasuhito Tanaka, Yusuke Sato, Michinori Kohara, Kyoko Tsukiyama-Kohara

**Affiliations:** a Transboundary Animal Diseases Centre, Joint Faculty of Veterinary Medicine, Kagoshima University, Kagoshima, Japan; b Department of Pharmacology & Toxicology, Faculty of Animal Science and Veterinary Medicine, Sher-e-Bangla Agricultural University, Dhaka, Bangladesh; c Department of Microbiology and Public Health, Faculty of Animal Science and Veterinary Medicine, Patuakhali Science and Technology University, Barishal, Bangladesh; d Laboratory for Molecular Design of Pharmaceutics, Faculty of Pharmaceutical Sciences, Hokkaido University, Hokkaido, Japan; e Department of Microbiology and Cell Biology, Tokyo Metropolitan Institute of Medical Science, Tokyo, Japan; f Department of Gastroenterology and Hepatology, Kumamoto University, Kumamoto, Japan; g Department of Viral Pathogenesis and Controls, National Institute of Global Health and Medicine, Japan Institute for Health Security, Chiba, Japan

**Keywords:** Hepatitis B virus, Lipid nanoparticles, CRISPR/Cas9, Delivery system

## Abstract

Patients with chronic hepatitis B virus (HBV) infection may benefit from clustered regularly interspaced short palindromic repeats (CRISPR)/CRISPR-associated protein 9 (Cas9)-based gene therapy. We previously identified a guide RNA (WJ11) that suppressed HBV replication *in vitro* and *in vivo*; however, we were unable to achieve delivery at clinically feasible doses *in vivo* using an adeno-associated virus (AAV) vector. Lipid nanoparticle (LNP)-based WJ11/Cas9 ribonucleoprotein-oligonucleotide complex delivery suppressed HBV replication by 2–3-fold more than did AAV-based delivery. In the present study, we investigated the HBV replication-suppressive effects of LNP/WJ11/Cas9 complexes after intravenous administration to persistently HBV genotype C-infected humanized chimeric mice. CL4H6 (ionizable lipid) LNPs were selected as the first candidate for WJ11/Cas9 delivery based on their reported high encapsulation efficiency; however, no significant anti-HBV effect was noted in serum or hepatic tissue. The ionizable lipid candidate CL4F11_ε-3 improved absolute serum HBV values to a certain degree but had no significant effect on hepatic HBV DNA or covalently closed circular (ccc)DNA levels. CL4F11_ζ-2 LNP/WJ11/Cas9, a new complex prepared through structural optimization of the ionizable lipid and heat treatment of WJ11, showed suppressive effect for serum viral load along with a reduction of hepatic HBV DNA, HBV cccDNA, HBsAg, and HBcrAg levels when compared with controls. Therefore, LNP-based delivery of this CRISPR/Cas9 formula holds promise for the treatment of chronic HBV infection.

## Highlights

• LNP-mediated *in vivo* delivery of gRNAs/Cas9 systems has been investigated.
• We investigated 3 LNP candidates for inhibition of HBV replication *in vivo*.
• CL4F11_ζ−2 LNP/WJ11-Cas9 showed significant suppression of serum HBV level.
• Heat treatment of WJ11 sgRNA may improve the efficacy of LNP-Cas9.

## 1 Introduction

Hepatitis B virus (HBV) infects more than one million people annually worldwide and causes chronic infection in 3.3 % of the global population (World Health Organization, 2025, Hepatitis B. Accessed on 27 November 2025. Available at: https://www.who.int/news-room/fact-sheets/detail/hepatitis-b.) posing a severe global health burden. The inflammation and fibrosis that accompany chronic hepatitis have been implicated in the development of cirrhosis and hepatocellular carcinoma (El-Serag HB, 2012). Considering this human health threat, the World Health Organization has set 2030 as the target date for the global elimination of HBV; however, according to current figures, achieving this will be challenging (World Health Organization, https://www.who.int/news-room/fact-sheets/detail/hepatitis-b). HBV can persist in host cells through the conversion of incoming relaxed circular DNA (Gomez-Moreno A and Ploss, A, 2024) into covalently closed circular DNA (cccDNA), which serves as a template for viral gene transcription. cccDNA transcripts evade immune surveillance and prevent conventional nucleoside/nucleotide analog-based therapies targeting HBV RNA transcriptase from achieving a functional cure (El-Serag HB, 2012; Yuen et al., 2018). HBV DNA integrated into the host genome in hepatocytes may continue to express viral proteins, which may elicit a sustained inflammatory immune response (Koumbi L, 2015; Yardeni D, Chang, KM and Ghany, MG, 2023). These phenomena pose a major obstacle to HBV control, necessitating new therapies.

Clustered regularly interspaced short palindromic repeats (CRISPR)/CRISPR-associated protein 9 (Cas9) genome editing-based therapies targeting HBV cccDNA and HBV DNA integrated in infected hepatocyte chromosomes may meet this need (Kayesh MEH et al., 2020; Li H et al., 2017; Lin SR et al., 2014; Stone D et al., 2021; White MK, Hu, W and Khalili, K, 2015). While *in vivo* studies have shown the promise of CRISPR/Cas9 systems for HBV treatment, the development of safe and effective delivery methods for successful clinical application remains a major challenge (Kayesh et al., 2020; Lin et al., 2014; Stone et al., 2021). We previously reported a CRISPR/Cas9 strategy using a guide RNA (gRNA; WJ11) to target HBV cccDNA (Kayesh MEH et al., 2020). WJ11 was selected from 16 candidate gRNAs based on its HBV-silencing efficiency. We used adeno-associated virus (AAV), a nonpathogenic parvovirus widely regarded as a strong vector candidate (Shen X, Storm, T and Kay, MA, 2007), to deliver the WJ11/Cas9 formula into infected hepatocytes. Experiments demonstrated that AAV2/WJ11/Cas9 suppressed HBV replication *in vitro* and *in vivo* (in humanized chimeric mice) and confirmed its anti-HBV effects in tree shrews, an immunocompetent animal model (Rashid MHO et al., 2025). However, the large amounts of AAV2/WJ11/Cas9 required for dosing in the *in vivo* experiments (*i.e.*, 10^12^ copies/animal for chimeric mice (Kayesh MEH et al., 2020) and 10^13^ copies/animal for tree shrews (Rashid MHO et al., 2025)) posed a barrier to its clinical application, necessitating enhancements in the WJ11/Cas9 delivery efficiency.

Concurrent with the developments in CRISPR/Cas9 editing, lipid nanoparticles (LNPs) have been developed as successful siRNA delivery systems (Suzuki Y et al., 2021). We previously used LNPs to deliver a CRISPR/Cas9 system and developed an LNP-based CRISPR/Cas9 ribonucleoprotein (RNP) delivery nanoplatform (Suzuki Y et al., 2021). *In vitro*, LNP/Cas9 suppressed HBV DNA and cccDNA by 2- and 4-fold more than did AAV/Cas9 (Suzuki Y et al., 2021).

In the present study, we optimized the LNP/WJ11/Cas9 formula to enhance its efficacy against HBV and allow lowering the amount required *in vivo* to contribute to the development of an *in vivo* delivery system for gene therapies targeting persistent HBV infection for clinical use.

## 2 Materials and methods

### 2.1 Preparation of Cas9 RNP-loaded CL4H6 LNPs

**Fig. 1:**
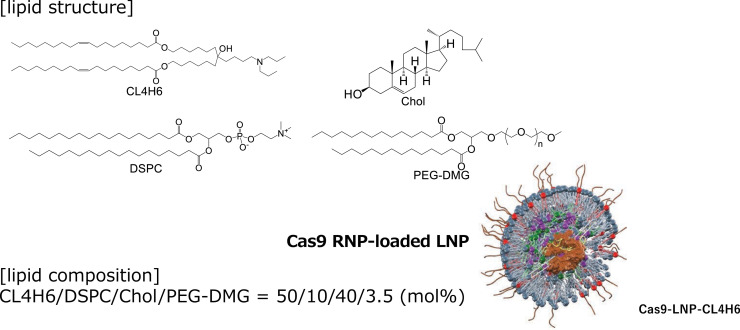
Lipid structure and composition of Cas9 (RNP)-loaded CL4H6 LNPs.

CL4H6, a pH-sensitive cationic lipid, was synthesized as described previously (Sato Y et al., 2019). CL4H6 LNPs for human liver tissues were prepared as described previously (Sato Y et al., 2021) (Fig. 1, Supplementary Table 1). The percentage encapsulation was 82.3–85.5 % (Supplementary Table 1). A solution of Cas protein (10 µM) (TrueCut Cas9 Protein; Thermo Fisher Scientific, Waltham, MA) was titrated into an equal volume of WJ11 single-guide RNA (sgRNA) (5′-ACUGUUCAAGCCUCCAAGCU-3′; nucleotide positions 1859–1878 in the HBV genome) (Kayesh MEH et al., 2020) or enhanced green fluorescent protein (GFP) sgRNA (5′-GAGCUGGACGGCGACGUAAA-3′; nucleotide positions 52–71 in the GFP gene) (Kayesh MEH et al., 2020) solution (10 µM) (Integrated DNA Technologies, Coralville, IA) under vigorous mixing to produce a 5 µM RNP solution. The RNP solution was mixed with an equal volume of a single-stranded oligodeoxynucleotide (ssODN) (Integrated DNA Technologies) solution (5 µM) to obtain 2.5 µM RNP-ssODN complexes (sgRNA, Cas9 ssODN mixture) (Onuma H, Sato, Y and Harashima, H, 2023). The target cleavage efficiency was 47.43 %, the encapsulation efficiency of the Cas9 protein was 84 %, and the delivery efficiency was 27 % (Onuma H, Sato, Y and Harashima, H, 2023). An ethanol solution containing 9-octadecenoic acid 1,1′-[7-[4-(dipropylamino)butyl]−7‑hydroxy-1,13-tridecanediyl] ester (CL4H6), 1,2-distearoyl-sn‑glycero-3-phosphatidylcholine (DSPC), chol, and polyethylene glycol-1,2-dimyristoyl-sn-glycerol-3-methoxypolyethylene glycol (PEG-DMG) at a molar ratio of 50:10:40:3.5 was prepared to achieve a total lipid concentration of 8 mM. The RNP-ssODN was diluted to 160 nM in 20 mM MES buffer (50 mM NaCl, pH 6.0). RNP-loaded LNPs were prepared by mixing the lipids in ethanol and RNP-ssODN in an aqueous solution using a polydimethylsiloxane-based iLiNP device (1 mm width) at a total flow rate of 2.0 mL/min (0.20 mL/min for the lipid solution and 1.80 mL/min for the RNP-ssODN solution), controlled using syringe pumps (Harvard Apparatus, Holliston, MA). The resultant LNP solution was dialyzed against 20 mM Tris–HCl buffer (pH 7.4) containing 9 % sucrose at 4 °C for at least 2 h using Spectra/Por 4 dialysis membranes (molecular weight cut-off 12–14 kDa; Spectrum Laboratories, Gardona, *CA*) and then concentrated via ultrafiltration using an Amicon Ultra-15 (molecular weight cut-off 100 kDa; Millipore, St. Louis, MO).

### 2.2 Preparation of Cas9 RNP-loaded CL4F11_ε−3 LNPs

The pH-sensitive cationic lipid CL4F11_ε−3 was synthesized as described previously (Onuma H et al., 2024). CL4F11_ε−3 LNPs were prepared using the same procedure as that used for CL4H6 LNPs except that CL4F11_ε−3 was used instead of CL4H6 as an ionizable lipid.

### 2.3 Preparation of Cas9 RNP-loaded CL4F11_ζ−2 LNPs

The pH-sensitive cationic lipid CL4F11_ζ−2 was synthesized as described previously (Onuma H et al., 2024). CL4F11_ζ−2 LNPs were prepared like CL4H6 LNPs except that CL4F11_ζ−2 was used instead of CL4H6 and WJ11 sgRNA (Kayesh MEH et al., 2020) was heated at 70 °C for 10 min to obtain monomeric secondary structure-fixed sgRNA before mixing with Cas9 protein. CL4F11_ζ−2 LNPs with *GFP*-targeting sgRNA (Kayesh MEH et al., 2020) were used as a control.

### 2.4 Mice and treatments

The humanized chimeric mice used in this study were homozygous albumin enhancer/promoter-driven urokinase-type plasminogen activator/severe combined immunodeficient (uPA/SCID) mice obtained from PhoenixBio (Higashi-Hiroshima, Japan). Mice of both sexes at 27–29 weeks of age were used in the experiments. UPA/SCID mice were transplanted with human hepatocytes on day 21 after birth. The average replacement ratio for human hepatocytes exceeded 80 %, as calculated previously (Mercer DF et al., 2001; Tateno C et al., 2015). Mice were infected with HBV genotype C_AT (2 × 10^5^ copies/mouse; GenBank AB246345) and administered with LNPs 2–4 months later (Nakagawa S et al., 2013). All animal experimental procedures were approved by the Institutional Animal Care and Use Committee of PhoenixBio Co., Ltd. (approval Nos. 2273, 2275, and 00458).

LNP/GFP/Cas9 and the relevant LNP/WJ11/Cas9 formulation were adjusted to 0.2 mg WJ11 gRNA/mL with Opti-MEM and injected via the tail vein (10 mL/kg) (*n* = 3). Blood (50 µL) was collected from each mouse through retro-orbital bleeding under anesthesia with ketamine hydrochloride (90 mg/kg) on days –1, 1, 3, 5, 7, 10, and 14, and serum was obtained by centrifugation at 10,000 rpm for 10 min. Serum HBV DNA titers were determined, and serum alanine aminotransferase (ALT), albumin, and HBV surface antigen (HBsAg) and HBV core-related antigen (HBcrAg) levels were measured at the indicated time points. On day 14, the mice were sacrificed under anesthesia and their livers were collected and stored at –80 °C until analysis.

### 2.5 Determination of the serum HBV DNA titer and characterization of HBV genome editing

Intrahepatic HBV DNA and cccDNA titers were determined in genomic DNA extracted from chimeric mouse liver tissues using the phenol-chloroform extraction method, as described previously (Kayesh MEH et al., 2020). HBV cccDNA was quantified using quantitative (q)PCR based on TaqMan chemistry using the forward primer HBV cccDNA 1519-S25 (5′-ACG GGG CGC ACC TCT CTT TAC GCG G-3′), reverse primer HBV cccDNA 1886-R25 (5′-CAA GGC ACA GCT TGG AGG CTT GAA C-3′), and TaqMan probe HBV cccDNA-1575 S26FT (5′−6-FAM-CCG TGT GCA CTT CGC TTC ACC TCT GC-TAMRA-3′). The cycling conditions were: 50 °C for 2 min, 95 °C for 10 min, and 53 cycles at 95 °C for 20 s and 65 °C for 1 min. qPCRs were run in a CFX Connect Real-Time PCR Detection System (Bio-Rad, Hercules, *CA*).

HBV genome editing was characterized using the T7 Endonuclease 1(E1) assay and PCR cloning. Total genomic DNA was extracted from the livers of humanized chimeric mice treated with LNP/GFP/Cas9 and the relevant LNP/WJ11/Cas9, amplified with HBV-794F (5′- CCTCTATTACCAATTTTCTTTTGTC-3′) and HBV-2051R (5′-GAGGAGAACAATGTTCCGGAGACT-3′) with Phusion polymerase (New England Biolabs, Ipswich, MA), and digested with T7E1 (New England Biolabs), as described previously (Kayesh MEH et al., 2020). The HBV genome was amplified with HBV-794-F and HBV-2051R and Phusion polymerase (NEB), subcloned into Zero Blunt TOPO PCR cloning kit (Thermo Fisher Scientific), and sequenced by Eurofins Genomics (Tokyo, Japan).

### 2.6 Measurement of serum ALT, albumin, and HBsAg/HBcrAg levels

Serum ALT was measured using a Transnase Nissui (Nissui Pharmaceutical Co. Ltd., Tokyo, Japan). The data were standardized and are expressed in IU/L. Serum albumin was quantified using a Human Albumin (hAlb) ELISA Quantitation kit (Bethyl Laboratories) per the manufacturer’s instructions (Supplementary Table 2). The serum concentration was calculated in mg/mL using a standard curve prepared in advance. Mouse serum HBsAg levels were measured using a two-step sandwich assay with a fully automated chemiluminescent enzyme immunoassay system (Lumipulse, 1200; Fujirebio, Tokyo, Japan), as described previously (Shinkai N et al., 2013). Mouse serum HBcrAg levels were measured using a high-sensitivity HBcrAg assay, as described previously (Inoue T et al., 2021).

### 2.7 Statistical analysis

Data are presented as mean ± standard deviation (SD). Student’s *t*-test was used for comparisons between two groups and one-way ANOVA with Dunnett’s test was used for multiple group comparisons. GraphPad Prism (GraphPad Software, La Jolla, *CA*) was used for all analyses. Statistical significance was set at *P* < 0.05.

## 3 Results

### 3.1 Effects of WJ11/Cas9-loaded CL4H6 LNPs *in vivo*

**Fig. 2:**
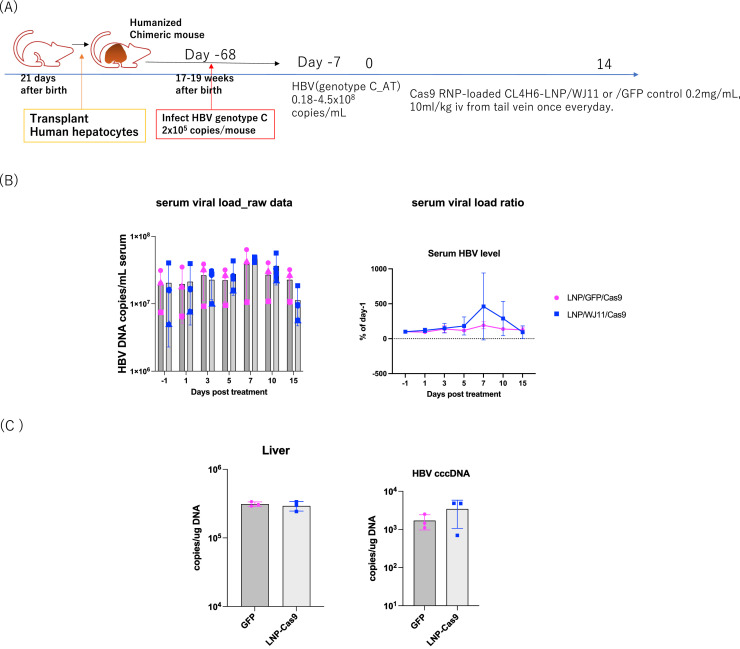
Evaluation of the efficacy of WJ11/Cas9 (RNP)-loaded CL4H6 LNPs against HBV infection in humanized chimeric mice. (**A**) Timeline of administration of Cas9 RNP-loaded CL4H6 LNPs to HBV genotype C_AT-infected humanized chimeric mice (*n* = 3). CL4H6-LNP/GFP/Cas9 (RNP)-treated humanized chimeric mice were used as controls (*n* = 3). (**B**) Serum HBV DNA levels as measured using qPCR (left) and serum HBV DNA ratios relative to day –1 (right). (**C**) Hepatic HBV DNA (upper) and cccDNA (lower) copies per genome DNA (μg) as quantified using qPCR. Vertical bars indicate SDs.

**Fig. 3:**
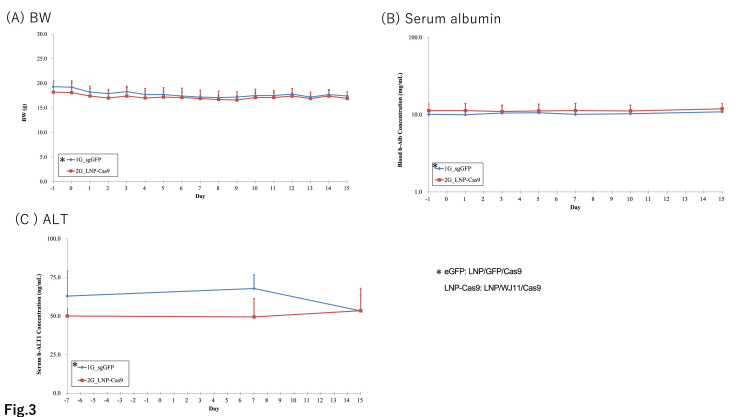
BW (**A**), serum albumin (**B**) and serum ALT (**C**) levels in humanized chimeric mice treated with WJ11/Cas9 (RNP)-loaded CL4H6 LNPs or LNP/GFP/Cas9 (control). Vertical bars indicate SDs.

We previously identified WJ11/Cas9 as a complex with potential efficacy against HBV (Kayesh et al., 2020); however, we experienced issues with the use of AAV for delivery. LNPs derived from the pH-sensitive cationic lipid CL4H6 reportedly have potential for siRNA delivery into hepatocytes (Sato Y et al., 2019, 2020). We investigated the efficacy of CL4H6 LNP loaded with pre-formed Cas9 protein-WJ11 gRNA RNPs (CL4H6 LNP loaded WJ11/Cas9) *in vivo* by using persistently HBV genotype C-infected humanized chimeric mice via the tail vein once daily for 14 days (*n* = 3) (Fig. 2A). Humanized chimeric mice were infected with HBV (genotype C_AT, 2 × 10^5^ copies/mouse) and incubated for 68 days. Serum HBV titers reached 0.18–4.5 × 10^8^ copies/mL on day 7 before LNP administration (Fig. 2A). Control mice (*n* = 3) were administered GFP/Cas9-loaded LNPs in the same manner. At sacrifice, blood and liver samples were collected to assess serum HBV DNA and hepatic HBV DNA and cccDNA copy numbers. LNP/WJ11/Cas9-treated mice showed no significant differences from LNP/GFP/Cas9-treated controls in terms of serum HBV DNA (Fig. 2B), hepatic HBV DNA, or hepatic cccDNA copy numbers (Fig. 2C). No significant differences were noted between LNP/WJ11/Cas9-treated mice and LNP/GFP/Cas9 controls in terms of body weight (BW) or serum albumin or ALT levels (Fig. 3). These findings indicated that CL4H6 LNP/WJ11/Cas9 showed no efficacy against HBV when delivered to humanized chimeric mice.

### 3.2 Effects of WJ11/Cas9 -loaded CL4F11_ε−3 LNPs *in vivo*

**Fig. 4:**
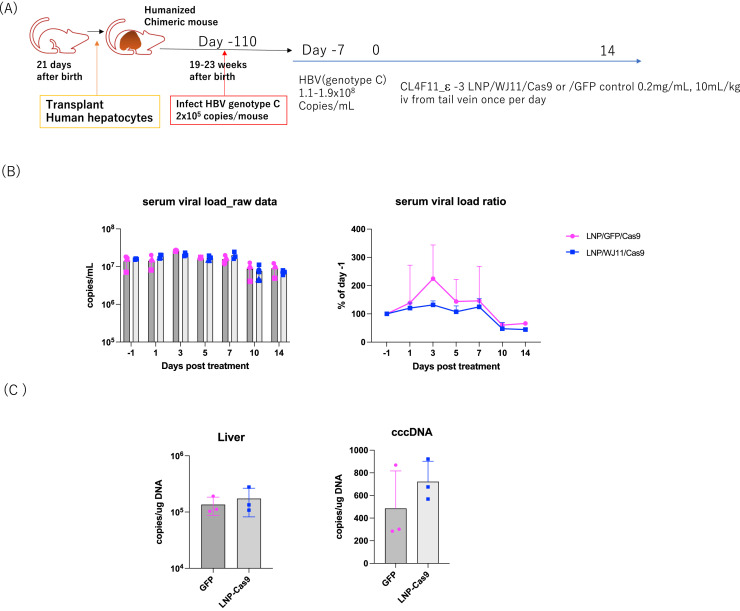
Evaluation of the efficacy of WJ11/Cas9 (RNP)-loaded CL4F11_ε−3 LNPs against HBV infection in humanized chimeric mice. (**A**) Timeline of administration of WJ11/Cas9 (RNP)-loaded CL4F11_ε−3 LNPs to HBV genotype C_AT-infected humanized chimeric mice (*n* = 3). LNP/GFP/Cas9-treated mice were used as controls (*n* = 3). (**B**) Serum HBV DNA levels as measured using qPCR (left) and serum HBV DNA ratios relative to day –1 (right). (**C**) Hepatic HBV DNA (upper) and cccDNA (lower) copies per genome DNA (μg) as quantified using qPCR. Vertical bars are SDs.

**Fig. 5:**
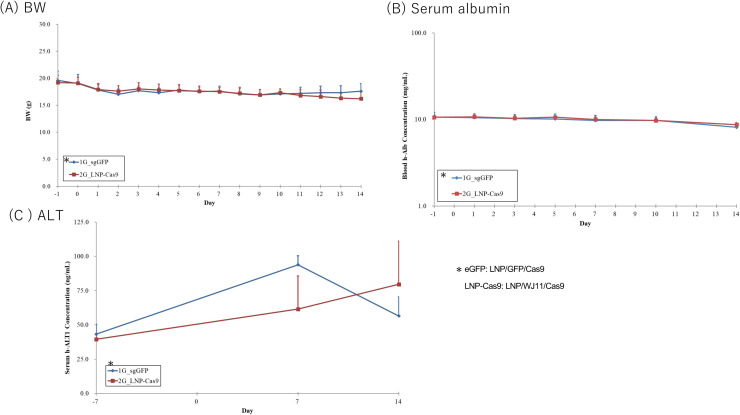
BW (**A**), serum albumin (**B**), and serum ALT(**C**) of humanized chimeric mice treated with WJ11/Cas9 (RNP)-loaded CL4F11_ε−3 LNPs or LNP/GFP/Cas9. Vertical bars are SDs.

As WJ11/Cas9 showed no effect when delivered with CL4H6 LNPs, we next evaluated delivery with a structurally modified cationic lipid, CL4F11_ε−3, which reportedly induced Fluc expression at approximately 10^8^ relative light units/mg protein in mouse liver and enhanced the delivery of gRNAs targeting liver specific enzyme transthyretin (TTR) and Cas9 RNAs, silencing serum TTR by >90 % at a 2 mg/kg dose (Onuma H et al., 2024). To investigate the efficacy of CL4F11_ε−3 LNP/WJ11/Cas9 *in vivo*, we administered WJ11/Cas9-loaded CL4F11_ε−3 LNPs to persistently (110 days) HBV genotype C-infected humanized chimeric mice (*n* = 3; serum HBV load on day 7 before LNP administration: 1.1–1.9 × 10^8^ copies/mL) once daily for 14 days (Fig. 4A). Although serum HBV DNA levels were <50 % of the viral load before treatment (day –1) in LNP-Cas9-treated mice after 14 days of treatment, we found no significant differences with LNP/GFP/Cas9 control-treated mice in serum HBV DNA copy number or level throughout the 14-day treatment period (Fig. 4B). At the end of the treatment period (day 14), HBV DNA and cccDNA copy numbers were higher in LNP/WJ11/Cas9-treated mice than in controls. No significant differences were noted between LNP/WJ11/Cas9-treated mice and controls in terms of BW (Fig. 5A) or serum albumin (Fig. 5B), although BW was slightly reduced in LNP/WJ11/Cas9-treated mice at the end of the treatment period. The serum ALT level linearly increased in LNP/WJ11/Cas9-treated mice throughout the 14-day treatment period, whereas in LNP/GFP/Cas9-treated control mice, it decreased from day 7 to day 14 (Fig. 5C).

### 3.3 Effects of WJ11/Cas9-loaded CL4F11_ζ−2 LNPs *in vivo*

**Fig. 6:**
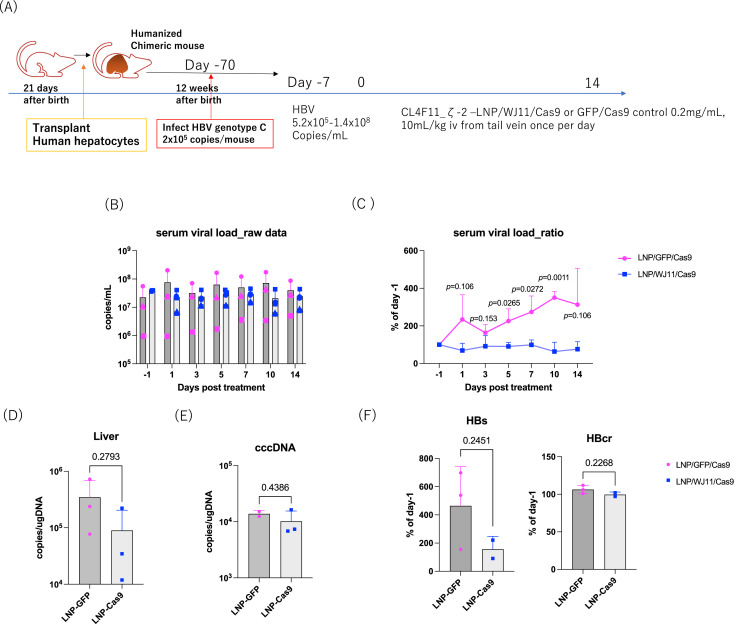
Evaluation of the efficacy of WJ11/Cas9 (RNP)-loaded CL4F11_ζ−2 LNPs against HBV infection in humanized chimeric mice. (**A**) Schematic representation of the time line of administration of WJ11/Cas9 (RNP)-loaded CL4F11_ζ−2 LNPs to HBV genotype C_AT infected humanized chimeric mice (*n* = 3). GFP/Cas9(RNP)-loaded LNP-treated humanized chimeric mice were used as controls (*n* = 3). (**B**) Serum HBV DNA levels as measured using qPCR (left) and (**C**) serum HBV DNA ratios relative to day –1 (right). (**D**) Hepatic HBV DNA and (**E**) cccDNA copies per genome DNA (μg) as quantified using qPCR. (**F**) Serum HBsAg and HBcrAg levels. Vertical bars are SDs.

**Fig. 7:**
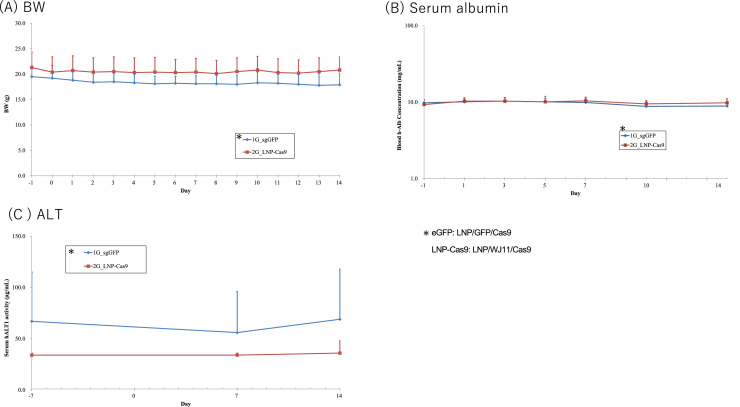
Body weight (**A**), serum albumin (**B**), and serum ALT (**C**) in humanized chimeric mice treated with GFP or WJ11/Cas9 (RNP)-loaded CL4F11_ζ−2 LNPs. Vertical bars are SDs.

As WJ11/Cas9 did not exert a significant effect even with the modified LNPs, we tested a cationic lipid with yet another different structural modification, CL4F11_ζ−2, considering its reported transduction efficiency in human liver tissue and rapid biodegradation, with a 90 % silencing effect at a lower dose of 0.75 mg/kg (Onuma H et al., 2024). Furthermore, we heat-treated WJ11 gRNA based on reports that such treatment increased the encapsulation efficiency of Cas9 RNP (LeBlanc C et al., 2018) (data not shown, Shimizu et al., accepted). To investigate the efficacy of CL4F11_ζ−2 LNP/WJ11/Cas9 *in vivo*, we administered WJ11/Cas9-loaded CL4F11_ζ−2 LNPs to persistently HBV genotype C-infected humanized chimeric mice (*n* = 3) once daily for 14 days (Fig. 6A, B). The HBV infection period was 70 days, and the serum HBV load reached 5.2 × 10^5^ to 1.4 × 10^8^ copies/mL on day 7 before LNP administration (Fig. 6A). CL4F11_ζ−2 LNP/WJ11/Cas9-treated chimeric mice showed reduced serum HBV levels when compared with LNP/GFP/Cas9-treated control mice throughout the treatment period, with significant reductions on days 5, 7, and 10 (*p* < 0.05, Fig. 6C). In addition, CL4F11_ζ−2 LNP/WJ11/Cas9-treated mice showed slightly lower hepatic HBV DNA and cccDNA copy numbers (Fig. 6D, E) and serum HBsAg and HBcrAg levels (Fig. 6F) on day 14, although the differences were not statistically significant. BW (Fig. 7A) and serum albumin (Fig. 7B) and ALT (Fig. 7C) levels remained comparable to the pre-treatment levels.

**Fig. 8:**
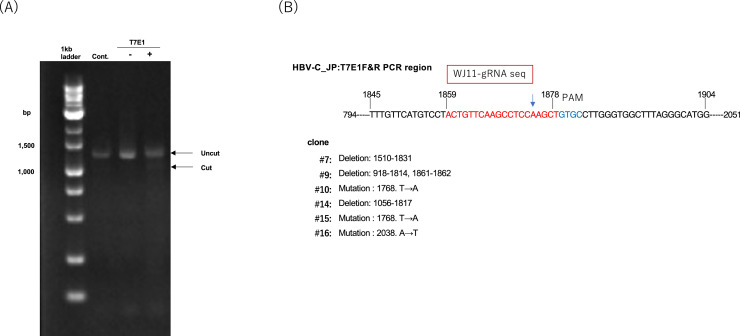
Characterization of HBV genome editing using the T7E1 assay (**A**) and sequencing (**B**). (**A)** HBV genomic DNA was amplified from the livers of chimeric mice treated with CL4F11_ζ−2 LNP/GFP/Cas9 (control) or LNP/WJ11/Cas9. Samples were treated (+) or not (–) with T7E1 nuclease, as indicated. A 1-kb DNA ladder marker was included, along with uncut and T7E1-digested HBV DNA. (**B**) Summary of HBV genome editing in chimeric mouse DNA treated with WJ11/Cas9 (RNP)-loaded CL4F11_ζ−2 LNPs. Edited regions were cloned and sequenced to confirm genome modifications.

Finally, we evaluated the effect of LNP/WJ11/Cas9-loaded LNPs on HBV genome editing. A T7E1 assay targeting the HBV genome region (nucleotides 794–2051) performed on liver DNA from chimeric mice yielded DNA fragments of the expected size (∼1.2 kb) (Fig. 8A). The HBV genome (1.2 kb) was subsequently amplified, subcloned, and sequenced (Fig. 8B). Among the 13 HBV clones analyzed, we identified deletions in clones #7, #9, #14, as well as point mutations in clones #10, #15, and #16.

## 4 Discussion

In this study, we optimized a system for the *in vivo* delivery of WJ11/Cas9—a CRISPR/Cas9 formulation that had previously shown efficacy *in vitro* and *in vivo* (Kayesh et al., 2020) but proved difficult to deliver at a clinically feasible dose using an AAV vector—by using LNP technology, which previously showed higher delivery activity than AAV *in vitro* (Suzuki Y et al., 2021).

A key finding of this study is the identification of CL4F11_ζ−2 LNP/WJ11/Cas9 as a formulation that showed an anti-HBV effect *in vivo* in humanized chimeric mice. This formulation showed suppressive effect to serum HBV DNA, along with hepatic HBV DNA and cccDNA levels. These findings corroborate previous findings on the efficacy of WJ11/Cas9 when delivered with an AAV vector to humanized chimeric mice (Kayesh et al., 2020) and tree shrews (Rashid et al., 2025). The advantage of LNP-based delivery lies in the clinically feasible human equivalent dose. In the previous studies, AAV/WJ11/Cas9 was administered at 10^12^ copies/animal to mice or 10^13^ copies/animal to tree shrews. BW-based dose conversion (considering an average BW of 150 g for tree shrews and 50 kg for humans) yields a human equivalent dose 10^15^ AAV-WJ11 for clinical administration targeting the liver. In contrast, the human equivalent dose of CL4F11_ζ−2 LNP/WJ11/Cas9 (0.2 mg/mL, 10 mL/kg) translates to 100 mg/patient, suggesting the potential for effective clinical delivery of this CRISPR/Cas9-based therapy targeting HBV-infected hepatocytes.

While AAVs are considered promising vectors for human gene therapy delivery, LNPs may have several potential advantages, including reduced immunogenicity, fewer off-target effects, and the avoidance of the generation of truncated Cas proteins. In the present study, they were an attractive choice mainly because of their reported superior targeting. LNP delivery with the cationic lipid CL4F11 was regarded as a promising option based on previous reports of efficient delivery *in vivo* to human hepatocytes (Hashiba et al., 2023) and spleen (Hashiba K et al., 2024). However, we were not able to replicate the anti-HBV effect of WJ11/Cas9 when using non-optimized CL4H6 LNPs. Interestingly, CL4H6 has greater tropism for the spleen than for the liver, depending on the formulation conditions (Sato Y et al., 2019). Therefore, further optimization of the lipid source may be required for the liver.

CL4F11 is an alternative LNP to CL4H6. Branched scaffolds derived from CL4F11 reportedly enhanced gRNA targeting of TTR, with CL4F11_ε−3 LNPs showing superior inhibition (Onuma H et al., 2024). We attempted WJ11/Cas9 delivery with CL4F11_ε−3 LNPs but were unable to demonstrate any significant effect in mice, although the difference in the absolute serum HBV load appeared marked at one time point. Given the previous report on CL4F11_ε−3 LNPs, we consider that this LNP may have the potential for inhibiting gene expression in the cytoplasm but not nuclear HBV replication.

CL4F11_ζ−2 LNPs were regarded as promising candidates because of their reported superior inhibition rate of over 90 % for TTR at 0.75 mg/kg, applicability to liver-specific genes, and biodegradability (Onuma H et al., 2024). RNP-loaded ζ LNPs may be optimal for delivering CRISPR/Cas9 products owing to their flower-like structure (Cheng MHY et al., 2023). However, the advantage of CL4F11_ζ−2 LNPs over other LNP formulations should be investigated in more detail in future studies, especially considering that WJ11 sgRNA was heat-treated in the present study.

Notably, among all optimizations attempted, we achieved the best results after heat treatment of the WJ11 sgRNA. Heat treatment establishes sgRNA as a homogenous monomer, increases the yield of monomeric Cas9 RNP, and improves the encapsulation and gene-editing efficiencies (LeBlanc C et al., 2018). Future studies to increase the sgRNA administration amount would help to efficiently suppress HBV replication in liver tissues. Taken together, these findings suggest that a highly optimized and high amount of LNP-Cas9 is required to suppress HBV replication in liver tissues because of the complex nature of the HBV replication cycle in cells (Ezzikouri S et al., 2014).

The LNP treatment used in this study appeared to have minimal adverse effects in humanized chimeric mice, as evidenced by no mortality, stable or increasing BW and consistent levels of human ALT, a key indicator of liver function. Further evaluation, including mouse ALT levels, blood chemistry, and histochemical analysis, will strengthen the safety of these LNP formulations.

The results of this study suggest that LNP-WJ11/Cas9 can be administered repeatedly, indicating its potential for clinical application. However, the efficiency of HBV DNA (including cccDNA) and antigen clearance in liver tissue remained low. Further optimization of the delivery system and administration protocol are essential to achieve complete elimination of HBV from the body. Additionally, the high cost of humanized chimeric mice and variations in serum HBV loads limited the number of animals used, representing a limitation of this study. The development of more cost-effective animal models is therefore required for future research.

In conclusion, CL4F11_ζ−2 LNPs show promise for the delivery of CRISPR/Cas9 products such as WJ11/Cas9 to human hepatocytes *in vivo* as a potential gene therapy approach for HBV. Further optimization would be an important step forward in HBV research and may contribute to the development of functional cure aimed at reducing or eliminating cccDNA.

## Ethics statement

This study was carried out in strict accordance with the Guidelines for Animal Experimentation of the Japanese Association for Laboratory Animal Science and the recommendations in the Guide for the Care and Use of Laboratory Animals of the National Institutes of Health. All protocols were approved by the Regional Ethics Committees.

## Funding

This work was supported by grants from the Japan Agency for Medical Research and Development (Nos. 25fk0310544s0301, 24fk0310515s0203, 23fk0310515s0202, and 22fk0310515s0201), Ministry of Health, Labour and Welfare, Japan.

## CRediT authorship contribution statement

**Rupaly Akhter:** Writing – original draft, Methodology, Data curation. **Bouchra Kitab:** Writing – review & editing, Investigation, Writing – original draft, Methodology, Data curation. **Mohammad Enamul Hoque Kayesh:** Writing – review & editing, Methodology, Investigation, Data curation. **Rina Shimizu:** Methodology, Data curation. **Haruno Onuma:** Methodology, Data curation. **Naoki Yamamoto:** Methodology, Data curation. **Shintaro Ogawa:** Methodology, Data curation. **Masaya Sugiyama:** Data curation. **Yasuhito Tanaka:** Supervision, Data curation. **Yusuke Sato:** Writing – review & editing, Validation, Methodology, Data curation. **Michinori Kohara:** Writing – review & editing, Supervision, Methodology, Investigation, Data curation. **Kyoko Tsukiyama-Kohara:** Writing – review & editing, Writing – original draft, Supervision, Investigation, Funding acquisition, Data curation, Conceptualization.

## Declaration of competing interest

The authors declare that they have no known competing financial interests.

## Data Availability

Data will be made available on request.

## Appendix Supplementary materials

Image, application 1

Image, application 2
