# Supplementary material for: Optimization of lipid nanoparticles loaded with ribonucleoprotein-oligonucleotide complexes for in vivo delivery of a CRISPR/Cas9 system targeting hepatitis B virus
Source: Virus Res. 2025 Dec 24;363:199682. doi: 10.1016/j.virusres.2025.199682 (PMC12811683; doi:10.1016/j.virusres.2025.199682)
Supplement: Supplementary file 1 [file mmc1.docx]

Supplementary Table 1. Physical properties of Cas9 RNP-loaded LNP with lipid of CL4H6

| LNP | | Enclosed (%) | Av.diameter ζ (nm) | PdI | Electric potential  Ζ (mV) | RNP (µg/µL) |
| --- | --- | --- | --- | --- | --- | --- |
| CL4H6 | WJ11 | 84 | 122 | 0.19 | 3.3 | 0.200 |
|  | eGFP | 85 | 119 | 0.18 | 3.2 | 0.200 |

WJ11 76.3 153 0.08 3.1 0.200

CL4F11_ε-3

eGFP 71.5 149 0.06 2.8 0.200

_____________________________________________________________________________________________________________________________

WJ11 83.8 122 0.19 3.3 0.462

CL4F11_ζ-2

eGFP 84.7 119 0.17 3.2 0.431

_____________________________________________________________________________________________________________
