# Supplementary material for: Optimization of lipid nanoparticles loaded with ribonucleoprotein-oligonucleotide complexes for in vivo delivery of a CRISPR/Cas9 system targeting hepatitis B virus
Source: Virus Res. 2025 Dec 24;363:199682. doi: 10.1016/j.virusres.2025.199682 (PMC12811683; doi:10.1016/j.virusres.2025.199682)
Supplement: Supplementary file 2 [file mmc2.docx]

|  | Supplementary Table2-1：Serum hAlb in humanized chimeric mice* | | | | | | | | | | | |
| --- | --- | --- | --- | --- | --- | --- | --- | --- | --- | --- | --- | --- |
|  | Mouse No. | Day | | | | | | | Minimum value during experiment | | |  |
| Treatment group |  | **−1** | 1 | 3 | 5 | 7 | 10 | 14 | Day | Value  (mg/mL) | Ratio to initial value_1)_ |  |
| CL4H6-LNP/GFP/Cas9 | 101 | 10.1 | 10.5 | 10.8 | 11.3 | 10.3 | 10.9 | 11.4 | −1 | 10.1 | 100 |  |
|  | 102 | 10.7 | 10.6 | 11.1 | 11.1 | 10.8 | 11.3 | 11.2 | 1 | 10.6 | 99 |  |
|  | 103 | 9.4 | 8.8 | 9.6 | 9.5 | 9.1 | 8.8 | 10.2 | 1, 10 | 8.8 | 94 |  |
|  | average | 10.1 | 10.0 | 10.5 | 10.6 | 10.1 | 10.3 | 10.9 | 1 | 10.0 | 99 |  |
|  | SD | 0.7 | 1.0 | 0.8 | 1.0 | 0.9 | 1.3 | 0.6 |  |  |  |  |
| CL4H6-LNP/WJ11/Cas9 | 201 | 12.2 | 12.5 | 12.3 | 12.9 | 13.5 | 12.4 | 13.0 | −1 | 12.2 | 100 |  |
|  | 202 | 13.3 | 13.1 | 12.4 | 12.4 | 12.1 | 12.4 | 13.2 | 7 | 12.1 | 91 |  |
|  | 203 | 8.5 | 8.2 | 8.4 | 8.4 | 8.3 | 8.8 | 9.4 | 1 | 8.2 | 96 |  |
|  | average | 11.3 | 11.3 | 11.0 | 11.2 | 11.3 | 11.2 | 11.9 | 3 | 11.0 | 97 |  |
|  | SD | 2.5 | 2.7 | 2.3 | 2.5 | 2.7 | 2.1 | 2.1 |  |  |  |  |
|  |  | | | | | | | | | | |  |
|  | (%)^1)^ | | | | | | | | | | |  |
|  | *mg/mL | | | | | | | | | | |  |
|  | Supplementary Table 2-2：serum hAlb in humanized chimeric mice* | | | | | | | | | | | |
|  | Mouse number | Day | | | | | | | Minimum value during experiment | | |  |
| Treatment group |  | **−1** | 1 | 3 | 5 | 7 | 10 | 14 | Day | Value  (mg/mL) | Ratio to initial value _1)_ |  |
| CL4F11_ε-3 LNP/GFP/Cas9 | 101 | 9.4 | 9.5 | 9.2 | 9.0 | 8.6 | 8.8 | 7.6 | 14 | 7.6 | 81 |  |
|  | 102 | 12.1 | 11.7 | 11.6 | 11.7 | 11.3 | 10.7 | 9.2 | 14 | 9.2 | 76 |  |
|  | 103 | 10.2 | 10.3 | 9.8 | 9.6 | 9.3 | 9.6 | 7.6 | 14 | 7.6 | 75 |  |
|  | average | 10.6 | 10.5 | 10.2 | 10.1 | 9.7 | 9.7 | 8.1 | 14 | 8.1 | 76 |  |
|  | SD | 1.4 | 1.1 | 1.2 | 1.4 | 1.4 | 1.0 | 0.9 |  |  |  |  |
| CL4F11_ε-3 LNP/WJ11/Cas9 | 201 | 11.3 | 11.4 | 10.9 | 10.7 | 10.7 | 10.3 | 9.2 | 14 | 9.2 | 81 |  |
|  | 202 | 10.0 | 10.3 | 9.6 | 10.7 | 9.6 | 9.1 | 8.4 | 14 | 8.4 | 84 |  |
|  | 203 | 10.6 | 10.5 | 10.4 | 10.3 | 9.8 | 9.8 | 8.4 | 14 | 8.4 | 79 |  |
|  | average | 10.6 | 10.7 | 10.3 | 10.6 | 10.0 | 9.7 | 8.7 | 14 | 8.7 | 82 |  |
|  | SD | 0.7 | 0.6 | 0.7 | 0.2 | 0.6 | 0.6 | 0.5 |  |  |  |  |
|  |  | | | | | | | | | | |  |
|  | (%)^1)^ | | | | | | | | | | |  |
|  | *mg/mL | | | | | | | | | | |  |
|  |  | | | | | | | | | | |  |

| Supplementary Table 2-2：serum hAlb in humanized chimeric mice* | | | | | | | | | | | |
| --- | --- | --- | --- | --- | --- | --- | --- | --- | --- | --- | --- |
| Treatment group | Mouse No. | Day | | | | | | | Minimum value during experiment | | |
|  |  | **−1** | 1 | 3 | 5 | 7 | 10 | 14 | Day | Value  (mg/mL) | Ratio to initial value _1)_ |
| CL4F11_ζ-3 LNP/GFP/Cas9 | 101 | 10.6 | 11.0 | 11.2 | 11.9 | 11.5 | 10.3 | 10.9 | 10 | 10.3 | 97 |
|  | 102 | 8.6 | 8.7 | 9.0 | 8.4 | 8.9 | 8.4 | 8.8 | 5, 10 | 8.4 | 98 |
|  | 103 | 10.1 | 10.7 | 10.6 | 9.9 | 9.2 | 7.6 | 7.1 | 14 | 7.1 | 70 |
|  | average | 9.8 | 10.1 | 10.3 | 10.1 | 9.9 | 8.8 | 8.9 | 10 | 8.8 | 90 |
|  | SD | 1.0 | 1.3 | 1.1 | 1.8 | 1.4 | 1.4 | 1.9 |  |  |  |
| CL4F11_ζ-3 LNP/WJ11/Cas9 | 201 | 8.8 | 9.4 | 9.1 | 9.0 | 9.3 | 8.6 | 8.4 | 14 | 8.4 | 95 |
|  | 202 | 10.1 | 11.1 | 11.4 | 11.0 | 11.7 | 10.5 | 11.1 | −1 | 10.1 | 100 |
|  | 203 | 9.0 | 10.5 | 10.4 | 10.3 | 10.3 | 9.5 | 9.9 | −1 | 9.0 | 100 |
|  | average | 9.3 | 10.3 | 10.3 | 10.1 | 10.4 | 9.5 | 9.8 | −1 | 9.3 | 100 |
|  | SD | 0.7 | 0.9 | 1.2 | 1.0 | 1.2 | 1.0 | 1.4 |  |  |  |
| 1)： | (%) | | | | | | | | | | |
| *： | mg/mL | | | | | | | | | | |
|  |  | | | | | | | | | | |
